# Are High Frequency Oscillations in Scalp EEG Related to Age?

**DOI:** 10.3389/fneur.2021.722657

**Published:** 2022-01-27

**Authors:** Philipp Franz Windhager, Adrian V. Marcu, Eugen Trinka, Arne Bathke, Yvonne Höller

**Affiliations:** ^1^Department of Neurology, Christian-Doppler Medical Centre and Centre for Cognitive Neuroscience, Paracelsus Medical University, Salzburg, Austria; ^2^Neuroscience Institute, Christian Doppler University Hospital, Paracelsus Medical University, Salzburg, Austria; ^3^Department of Mathematics, Paris Lodron University Salzburg, Salzburg, Austria; ^4^Faculty of Psychology, University of Akureyri, Akureyri, Iceland

**Keywords:** high frequency oscillation, electroencephalogram, scalp-EEG, HD-EEG, epilepsy

## Abstract

**Background:**

High-frequency oscillations (HFOs) have received much attention in recent years, particularly in the clinical context. In addition to their application as a marker for pathological changes in patients with epilepsy, HFOs have also been brought into context with several physiological mechanisms. Furthermore, recent studies reported a relation between an increase of HFO rate and age in invasive EEG recordings. The present study aimed to investigate whether this relation can be replicated in scalp-EEG.

**Methods:**

We recorded high-density EEG from 11 epilepsy patients at rest as well as during motor performance. Manual detection of HFOs was performed by two independent raters following a standardized protocol. Patients were grouped by age into younger (<25 years) and older (>50 years) participants.

**Results:**

No significant difference of HFO-rates was found between groups [*U* = 10.5, *p* = 0.429, *r* = 0.3].

**Conclusions:**

Lack of replicability of the age effect of HFOs may be due to the local propagation patterns of age-related HFOs occurring in deep structures. However, limitations such as small sample size, decreased signal-to-noise ratio as compared to invasive recordings, as well as HFO-mimicking artifacts must be considered.

## Introduction

In the last 2 decades high frequency oscillations (HFOs) have been studied extensively (1, 2). These HFOs are considered as oscillatory field potentials in the gamma- or high gamma band standing out of the oscillatory background activity showing a regular morphology that can be classified as ripples (80–250 Hz) and fast ripples (>250 Hz) (3). Bragin et al. (4) found that HFOs, in particular fast ripples, are typical for the epileptogenic region and may be a correlate of pathological changes leading to hypersynchronously bursting pyramid cells (5, 6). Therefore, HFOs are considered as a candidate marker for epilepsy (1) not only in adults but also in neonates (7) and children with refractory epilepsy (8).

Besides the assumed importance of HFOs in epilepsy diagnosis, earlier studies have shown that activity in this higher frequency range can also represent a physiological mechanism for synchronization of interneural firing not only of coherent neurons but also of neurons in spatial separated cortical areas (9–11). Further, HFOs recorded in both healthy and unhealthy patients can be seen during various cognitive processes such as memory-consolidation (12–14) motor-performance (15), and visual perception (16, 17). Thus, HFOs might not be displaying a genuine pathological characteristic in patients with neurological disease. However, the pathological relevance of this new biomarker may come into play when physiological mechanisms are disturbed.

What we know today about HFOs is largely based on empirical studies which investigated under what circumstances the phenomenon can be observed. Using invasive recordings as well as scalp EEG, scientists aimed to gather information about this promising biomarker in neuroscience (2, 18, 19). It is still a major challenge to identify potential indicators for a valid differentiation between pathological and physiological HFOs. By delineating particular characteristics like the background oscillatory activity (10), phase locking of HFOs and low frequencies recorded during sleep (20–22), spatial and signal characteristics (23) such as the amplitude (24), morphology (25), the occurrence in relation to an epileptic spike (26, 27), current research strives to contribute important information concerning the differentiation of physiological and pathological HFOs.

HFOs have been observed most often in invasive EEG recordings, using intracranial implanted macro- and micro-electrodes in patients with refractory focal epilepsy therefore undergoing presurgical examination (28). These studies reported a link between fast ripples and interictal spikes which are a common marker for identification of the seizure onset zone (SOZ). It would be advantageous to be able to examine HFOs in scalp EEG, in order to make this biomarker available also to patient populations without the necessity for an invasive recording. Because of the small amplitude and local propagation characteristics of HFOs it was highly debated whether HFOs can be detected in scalp EEG (16, 29). Scalp-EEG measures the summated activity of large populations of neocortical neurons and is much more prone to exogenous artifacts and noise. Local propagation patterns of HFOs (30) and their small size in relation to prominent muscle and movement artifacts in scalp EEG put the undertaking of detecting HFOs in the scalp into question. Recent work suggests that it is indeed possible (31, 32). It was suggested that a high-density (HD-EEG) would be advantageous over a conventional 10–20 montage; more specifically, by using HD-EEG, corresponding areas could be identified between scalp and invasive recordings (31).

Identification of HFOs in scalp-EEG is not straightforward (33, 34). First, the presumed pathological form of HFOs is likely to co-occur with physiological HFOs in scalp EEG (35) such that methods for the distinction of these phenomena are highly warranted. Second, it is crucial to increase the signal to noise ratio (36, 37), third, to use adequate spatial sampling (31), and, fourth, to reduce and correctly identify non-neural signals, so called artifacts, in order to allow application of scalp-based HFO analysis in clinical routine. Recent developments of custom-made low-noise amplifiers (34, 37) allow for a more accurate identification of HFOs in scalp EEG, which also correspond well with invasively recorded HFOs (34). However, the distinction of pathological from physiological HFOs in scalp EEG is a topic of its own merit, as these two phenomena are highly similar (33). A common approach is to compare HFOs in epileptic and non-epileptic EEG (33), but it is so far impossible to distinguish pathological from physiological HFOs on an individual case basis within a single recording. Nevertheless, because of their specificity scalp-HFOs bear a big potential to serve as interictal markers for epilepsy (38).

Scalp-EEG bears a further challenge, namely age. The EEG changes with age (39), and therefore it is also discussed as a factor that contributes to the development of epileptogenicity, due to a complex interaction between epilepsy and other diseases of the aging brain like stroke, dementia or traumatic brain injuries (40). From a structural view, the pathophysiology of temporal lobe epilepsy does resemble premature brain aging (41). As a consequence, the prevalence of active epilepsy increases with age (42) and might explain the high incidence of temporal lobe epilepsy in the elderly. Further investigations conducted by Tombini et al. (43) showing a link between the extent of neural degeneration resulting from diseases like Alzheimer's disease (AD) and epilepsy may be of interest since the number of HFO may predict the severity of epilepsy, therefore reflecting the extent of neural degeneration. Accordingly, HFO may also be used as a marker for the classification of neurodegenerative disorders such as AD in the future.

If we assume that the increase of HFOs in patients with epilepsy is due to structural changes resembling premature brain aging (41), it must be considered that HFO rate occurs to a greater extent in elderly patients independent of disease. Following this it can be hypothesized that a certain amount of HFO increase in elderly epilepsy patients is due to structural changes rather than simple pathological reasons. This implies that HFOs might not sensitively differentiate between aging effects and epileptogenic processes. Therefore, we claim that there is a need to understand how HFO rates change in elderly patients.

Previous research reported a link between age and HFO occurrence in invasive EEG (44). However, this finding was so far not replicated in scalp EEG. A recently conducted study by Cserpan et al. (45) found, that HFO activity in the ripple band is increased in a pediatric population of epilepsy patients compared to older subjects. They found that younger children showed a higher HFO compared to older children. Studies like these show that it is indispensable to estimate the contribution of age to the likelihood of HFO occurrence.

If older patients are more likely to exhibit HFOs regardless of epileptic pathology, HFOs may not be a reliable marker for epilepsy in this age group. The occurrence of high HFO rates in elderly patients with epilepsy might just be more diffusely related to physiologic than pathophysiologic changes that are a consequence of aging.

Using scalp-EEG we examined a possible relationship between age and HFO activity by comparing HFO rates between older and younger patients suffering from epilepsy as well as healthy controls.

## Materials and Methods

### Recruitment

We prospectively enrolled patients admitted to the Epilepsy Monitoring Unit (EMU) at the Department of Neurology, Christian-Doppler Medical Center Salzburg. Patients were admitted to the hospital for epilepsy clarification using video-EEG. Recruitment for the study was done one week prior to admission via phone. On the day of admission, patients who agreed to participate got informed about the purpose and possible side effects and signed informed consent prior to starting the experimental procedure.

### Ethics

The study was conducted in accordance with the recommendations of good clinical practice and was approved by the local ethics committee (Ethik-Kommission für das Bundesland Salzburg: E/1755, 415-E/1806/4-2014, initial approval on 30/03/2014, latest amendment on 11/07/2016).

### Participants

Between February 2018 and December 2020 48 participants had been enrolled to the study. For the analysis at hand only complete datasets of participants younger than 25 and older than 50 years with a sufficient signal to noise ratio (SNR) and minimal artificial distortions were included. We also excluded data from patients with extensive motor symptoms according to diagnosis. EEG segments with sufficient signal quality were exported. According to their age participants were assigned to one of two groups: < *25 Years* vs. > *50 Years*. In the < *25 Years* group we excluded 2 patients because final diagnosis made by the physician did not confirm an epileptic disease (1 male patient with non-epileptic psychosis and 1 female patient suffering migraine). In the > *50 years* group we excluded 3 patients because final diagnosis made by the physician did not confirm an epileptic disease (2 women with epileptiform discharges of unclear cause and 1 woman with restless-legs syndrome). Excluded patients served as controls. In patients labeled with “no final diagnosis” physicians where not absolutely sure about hemispheric localization of seizure onset zone.

11 Patients met all Criteria (n_ <25_ = 6; 4 Women; 2 Righthanded; M_ <25_ = 21.17 Years, SD_ <25_ = 2.14 Years; n_>50_ = 5; 2 Women; 2 Righthanded; M_>50_ = 57.2 Years, SD_>50_ = 3.83 Years). For a Detailed Overview of Patient Information see Table 1.

**Table 1:** Patient information.

**ID**	**Sex**	**Age**	**Hand**.	**AoO**	**Epilepsy**	**Seizure type**
<25 Years						
004	F	24	Right	5	TLE right	Focal aware and focal to bilateral tonic-clonic seizures
022	F	23	Left	20	MTLE right	Focal impaired awareness seizures
023	F	22	Right	3	PTME Left	Focal aware seizures; Focal impaired awareness seizures; Focal to bilateral tonic-clonic seizures
024	M	19	Right	4	OLE Right	Focal aware seizures; Focal impaired awareness seizures; Focal to bilateral tonic-clonic seizures
028	M	19	Right	5	FLE	Focal aware seizures; Focal impaired awareness motor seizures; Focal to bilateral tonic-clonic seizures
029	F	22	Left	20	NFD	Focal to bilateral tonic- clonic seizures
> 50 Years
002	M	56	Right	42	TLE left	Focal aware seizures
007	F	59	Right	59	TLE left	Focal impaired awareness seizures
037	M	64	Left	63	NFD	Focal to bilateral tonic- clonic seizures
039	F	54	Right	53	NFD	Speech impairment; cognitive restrictions; vertigo
042	M	55	Right	53	NFD	Focal to bilateral tonic- clonic seizures

*M, Male; F, Female; AoO, Age of Onset; Hand., Handedness; NFD, no final diagnosis; TLE, Temporal Lobe Epilepsy; MTLE, Mesial Temporal Lobe Epilepsy; PTME, Posttraumatic Multifocal Epilepsy; OLE, Occipital Lobe Epilepsy; FLE, Frontal Lobe Epilepsy*.

Regarding the control group, the study at hand used patients retrospectively diagnosed as “non-epileptic” by the physicians at the hosting institution. Since the under-25 age group contained only 2 and the over-50 age group contained only 3 subjects, controls were combined into a common control group (see Table 2). This results in a rather small sample (n_contr._= 5; 4 women; 5 righthanded; M_contr._ = 38.6 years, SD_contr._= 17.9 years). In future studies this should be considered and larger control groups should be recruited in order to distinguish physiological aging from epilepsy-associated processes and their relation to HFO activity.

**Table 2:** Control group.

**ID**	**Sex**	**Age**	**Hand**.	**AoO**	**No. of HFOs**
026	F	20	Right	5	4
027	M	18	Right	20	9
020	F	52	Right	42	0
043	F	52	Right	59	17
046	F	51	Right	63	0

*ID, Identification number; M, Male; F, Female; Hand., Handedness; AoO, Age of Onset; No. of HFOs, Number of HFOs*.

### Data Acquisition

Recording of high-density (HD) EEG was performed at the first day of hospitalization before starting the video-EEG monitoring. We decided to use HD-EEG, because previous studies reported a higher correspondence of areas with high ripple rates between intracranial EEG and HD-EEG compared to 10–20 EEG (31). A study conducted by Avigdor et al. (46) retrospectively analyzed EEG post-surgical recordings of patients with drug resistant epilepsy and found that the highest rates of HFOs can be found within the resected areas. Using both HD-EEG and the reduced 10–10 as well as the 10–20 EEG they showed that the detection of HFOs is even more accurate when conducted using HD-EEG and can be used for the identification of the epileptogenic zone (EZ.) They stated that an increase of localization accuracy of 40–60 percent can be achieved.

The experiment was performed using Presentation® software (Version 18.1, Neurobehavioral Systems, Inc., Berkeley, CA). HD-EEG recording was performed using a 256 channel EGI Hydrocel Net (Net Station Acquisition 5.0, Electrical geodesics, Inc.). Prior to the EEG recording, impedances of the electrodes were checked and kept within a range of 50 to 100 kΩ. Electrode number 43 was excluded in all recordings because of a hardware problem.

To cut out powerline noise a 50 Hz notch filter was applied. Sampling rate was 1 kHz, fulfilling Nyquist- criteria and a high pass filter of 0.1 Hz enabled elimination of low frequencies outside the spectrum of interest. In order to guarantee high data quality, patients were asked to intentionally generate artifacts during an initial calibration recording, which served as a reference for postprocessing artifact exclusion. Participants were asked to blink, raise their eyebrows, swallow and chatter their teeth several times in succession. To control for facial muscle activity, an electromyogram (EMG) was recorded from the patients' cheek. Participants were encouraged to move as few as possible between the test segments in order to minimize artificial distortion in the EEG.

### Experimental Procedure

Patients were seated in a comfortable chair. During the initial resting recording, patients were asked to sit as relaxed as possible with closed eyes for one minute. Next, a fingertapping task comprising 12 learning and 5 recall trials was conducted. Each of the trials lasted for 30 seconds and was separated by a short intertrial break of equal duration. The task used was the same as described by Gerner et al. (29). Participants were instructed to repeatedly type a five-digit sequence presented on the screen as fast and accurate as possible with their non-dominant hand. The task included a practice-, a learning-, and a recall-part. Following previous studies, showing a link between number of HFOs and neural learning processes in the somatomotoric cortex (5), we decided to analyze the learning part in the study at hand to maximize probability of event detection. During the intertrial breaks, participants were told to relax their fingers on the keyboard to minimize artificial activity. Prior to the experiments recall-phase, a resting phase of ten min was recorded. In total, the testing-procedure lasted for about 1.5 h.

### HFO Identification/Marking Procedure

**Figure 1:**
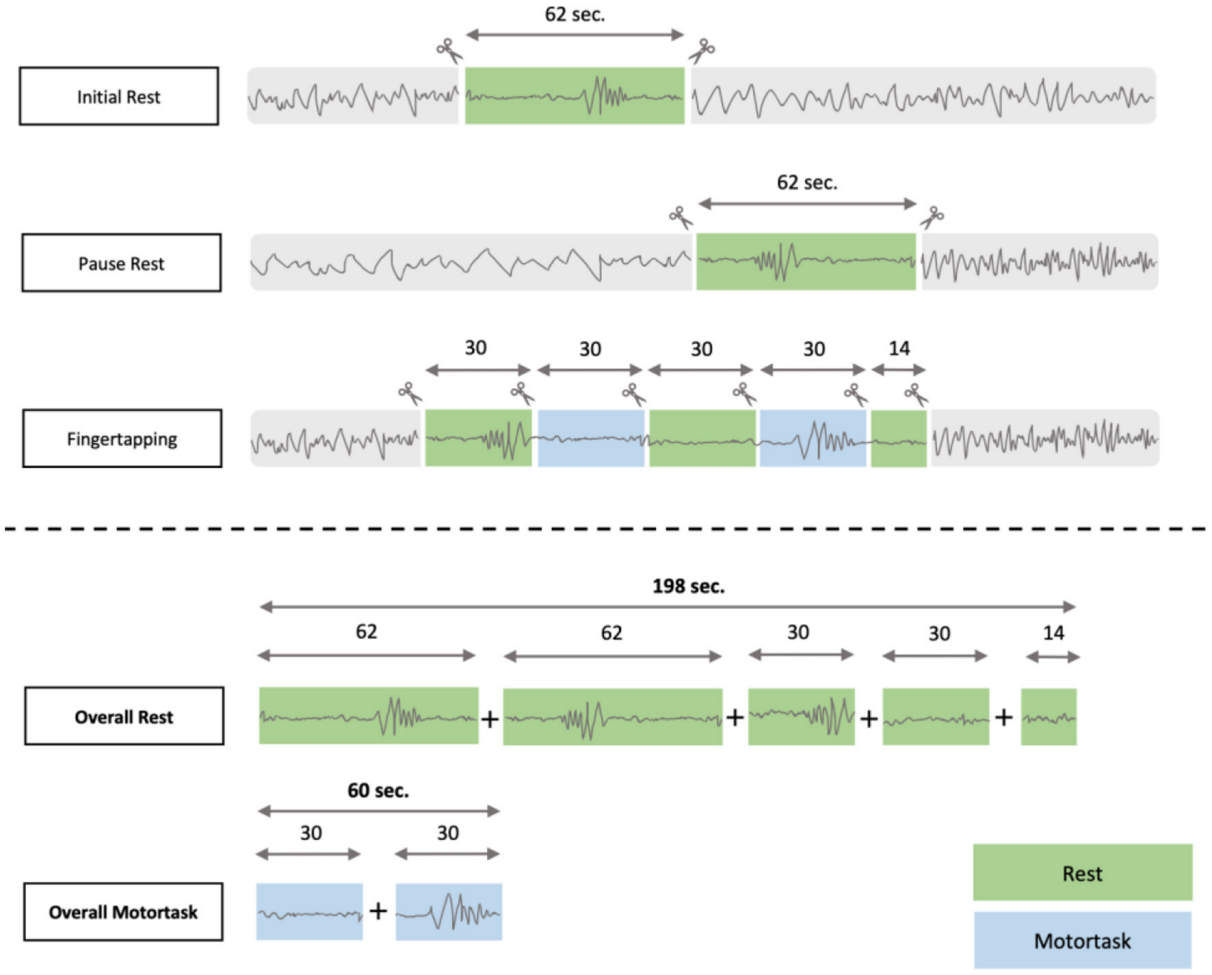
EEG- segmentation process: The overall time for resting was 198 seconds compared to 60 seconds of motortask related EEG segments.

The marking procedure to identify HFOs closely followed the procedure as presented in previously published research (19, 29). Data was analyzed using an in-house built software for HFO analysis called MEEGIPS (33). For the analysis we extracted EEG-segments with a maximal SNR and minimal artificial distortion of 198 seconds duration overall including initial rest, pause rest as well as the breaks between the single fingertapping segments (i-rest + p-rest + breaks of fingertapping). EEG-segments of 60 seconds duration were extracted from the learning-part of the fingertapping task (for composition of segments see Figure 1).

Segments were analyzed manually and independently by two experienced raters. HFO identification was blinded with respect to epilepsy- lateralization and diagnosis to minimize rater-bias. In a first step, the raters visually identified the events of interest independently. Due to several factors of manual analysis of scalp EEG in general and HD-EEG in special, interrater reliability was not available. Because of this, we decided to take into account all events marked by the two raters independently. For the final analysis, the detected events were reviewed by both raters until an agreement was reached regarding the classification of the specific events (ripple vs. unclear HFO vs. artifact).

**Figure 2:**
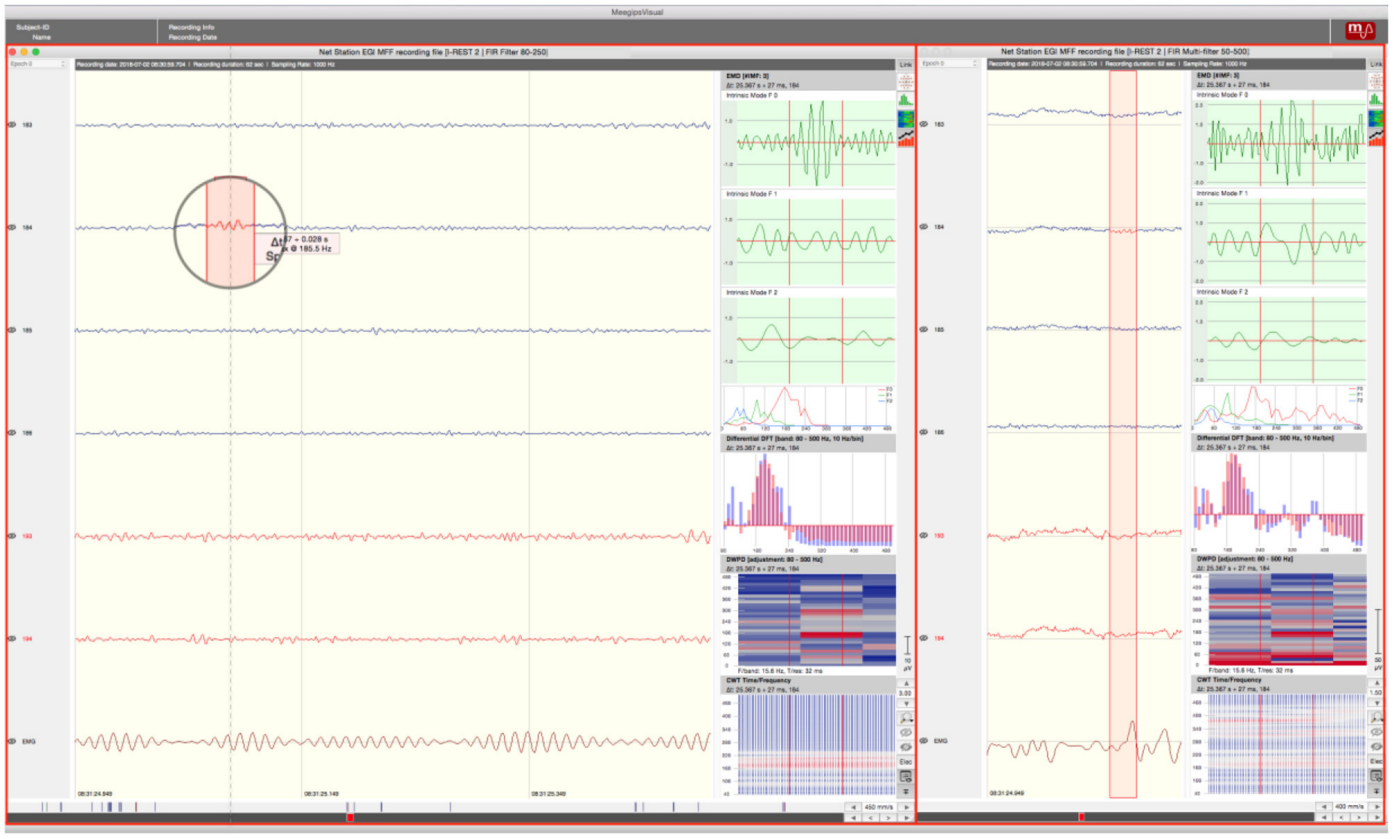
Screen Arrangement MEEGIPS: **Left side window** depicts FIR filtered data (80–250Hz); **Right side window** depicts multifiltered data (50–500Hz). An overlap between marked event (red window in circle) and EMG activity can be seen. Therefore this event would have been marked as an artifact. Red marked channels were excluded.

A total of 124 channels per segment were analyzed. Prior to HFO marking, two different sets of filters were applied to the data. A *finite impulse response filter* (FIR) labeled as *multifilter* with a frequency-range of 50–500 Hz was applied. We used this filter to remove the harmonics of line noise from the EEG-data and the FIR provides a higher constancy in phase-delay. The so-filtered data was obtained to enable identification of frequency activity typically seen in non-neural events like motor-dependent muscle movement. Afterwards, the same segment was filtered using an FIR in the gamma band with a frequency range of 80–250 Hz for the purpose of a detailed HFO analysis. Both versions of data (filtered at 50–500 Hz and at 80–250 Hz) were viewed simultaneously (see Figure 2).

The gamma-range filtered EEG-segment (80–250 Hz) was viewed with a time resolution of 450 mm/s, a timescale of 0.6 seconds per view and an amplitude scaling of 3,00 μV. Standard scaling with zero mean corrected and 50–500 Hz filtered data was placed on the right side of the desktop. Time resolution was 310 mm/s and amplitude scaling was 1,10 μV. Timescaling was 0.2 second per view.

Raters always analyzed groups of 6 channels simultaneously plus an additional EMG channel. Grouping of channels was based on spatial proximity to each other. Channels with low SNR or continuous artifacts were excluded. However, excluded channels were not blinded but merely marked with a red layer to serve as a reference for artifact identification (See Figure 2). According to Wang et al. (47) the ROI was defined over the central (C3, C4), the adjacent frontal (F3, Fz, F4) and parietal (P3, Pz, P4) region corresponding to the 10–20 montage of the scalp EEG. HFOs recorded during motor task performance were previously related to motor planning (48) and motor action (49). Therefore, the ROI should cover the premotor, motor and somatosensory cortices. For HFO analysis in the resting EEG, the ROI should cover a large region including the fronto-parietal default mode network (50). To minimize contamination effects in statistics due to false positives generated by muscle artifacts mimicking HFO's we previously excluded channels spatially related to cranial muscles (e.g. orbicularis oculi muscle, occipitofrontalis muscle, temporal muscle). The 124 channels were the same for all patients and were shared symmetrically between the hemispheres. Channels of interest were chosen relating to their corresponding areas associated with a higher activity during different tasks (48–50). Additionally, we excluded all channels that showed continuous artifacts, no signal at all as well as channels with poor signal to noise ratio. By doing so, we reduced the number of channels from 256 to 124. Events of interest (EoI) that correlated with eye blinks within a range of 200ms before and after the event were also excluded. To do so, we used some of the frontal electrodes of the HD-EEG net as electrooculographic electrodes.

**Figure 3:**
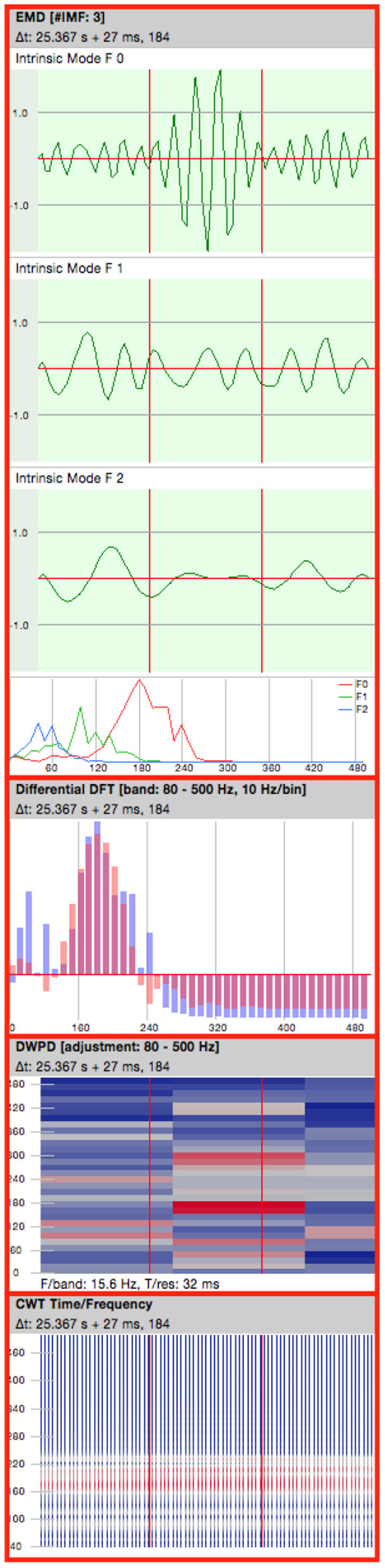
Analysis pane. EMD, Empirical Mode Decomposition (4 consecutive oscillations are shown); DFT, Differential Fourier Transform; DWPD, Discrete Wavelet Packet Decomposition; CWT, Continuous Wavelet Transform.

An analysis pane (Figure 3) with a set of configurable tools for evaluation of the selected EEG data was enabled for each of the displayed windows (33). The following representations were used for analysis of potential HFOs:

1. Empirical Mode Decomposition (EMD): Highpassfilter based on EMD (51).
2. Differential Fourier Transform (DFT).
3. Discrete Wavelet Packet Decomposition (DWPD): Signal-Power extraction over the segments using discrete time and frequency steps.
4. CWT: Continuous Wavelet Transform.

Events of interest (EoI) were marked by each rater and then classified using the information provided by the analysis pane. EoI's were classified into 3 categories, based on recent research investigating HFO-identification on the scalp EEG (29, 38, 52). Events consisting of at least 4 consecutive oscillations showing a regular morphology were considered as HFOs. The signals should stand out significantly from the background with respect to their amplitude and a superimposed activity should be visible in the multi-filtered raw data. A superimposed activity is an oscillation of high frequency, which “rides” on an oscillation of lower frequency.

Classification of events was performed as follows:

(i) Events showing an isolated high frequency activity in the high pass filtered EEG and a superimposed activity in the multifilter were considered as HFOs (ripple).
(ii) Events showing both, a high frequency activity in the high pass-filtered EEG as well as discrete “blob” in the DWPD however no superimposed activity in the raw data, were designated as unclear HFOs *(uHFO)*.
(iii) Events that did not fulfill the criteria of an isolated blob neither showed a superimposed activity in the raw data were considered as artifacts *(genArt)*. Activity showing a correlation between the observed EEG and the EMG activity were assumed to be triggered by muscle activity and also marked as artifacts.

Table 3 gives an overview about event classifications. An isolated blob shows at which frequency the oscillation has its maximum, i.e. where the signal power is located. Thus, an isolated blob is a quality mark that gives information about the “clarity” of the oscillation. When oscillations of various frequencies overlap the pattern in the DWPD looks more widespread. Artifacts show a broader representation in the frequency spectrum whereas HFO events appear as isolated “islands”. Thus, DWPD can be used to exclude artifacts and verify true events.

**Table 3:** Classification criteria and categories of events: Filtered: HFOs visible in High pass filtered data; Blob: isolated Blob visible in DWPD; Raw Data: superimposed activity visible in multi filtered raw data; Eventtype: High frequency oscillation (HFO); unclear high frequency oscillation (uHFO); generic Artifact (genArt).

**Filtered**	**Blob**	**Raw Data**	**Eventtype**
✓	✗	✓	*HFO (Ripple)*
✓	✓	✗	*uHFO*
✓	✗	✗	*genArt*

### Statistical Analysis

Statistical analysis aimed to answer the question whether there is a significant difference between the groups of young and elderly patients regarding mean HFO rates. Statistical analyses were carried out using the IBM SPSS Statistics Software (IBM Corp. Released 2011. IBM SPSS Statistics for Windows, Version 20.0. Armonk, NY: IBM Corp). Due to the small sample size, we conducted non-parametric tests. Rates of ripples and unclear HFOs identified during resting phases as well as during learning trials of the fingertapping task were calculated for each patient. The resting phases included the events identified during initial rest, pause rest, and the resting trials of the motor learning task. Because of the relatively small number of identified ripples, we also looked at the overall HFO rate, where in addition to ripples, we also included unclear HFOs (*HFO* = *ripples* + *uHFO*).

To test for significant group differences between younger and older patients, *Mann-Whitney tests* were carried out, comparing the ripple rates and the overall HFO rates both during the motor learning task and during resting phases. Because of multiple comparisons, all results were interpreted at the Bonferroni corrected level of significance *p* = 0.05/6 = 0.008.

Furthermore, we also conducted a semi-parametric repeated measures ANOVA with the factors age and condition to compare the detected HFOs during rest with those recorded during motor tasks.

We chose this method that only requires metric data but allows for non-normality and variance heterogeneity (53). This method is implemented in the function RM of the R-package MANOVA.RM (54). We used it with the parametric bootstrap with 1000 iterations. The parametric bootstrap showed the most favorable performance in unbalanced designs.

**Figure 4:**
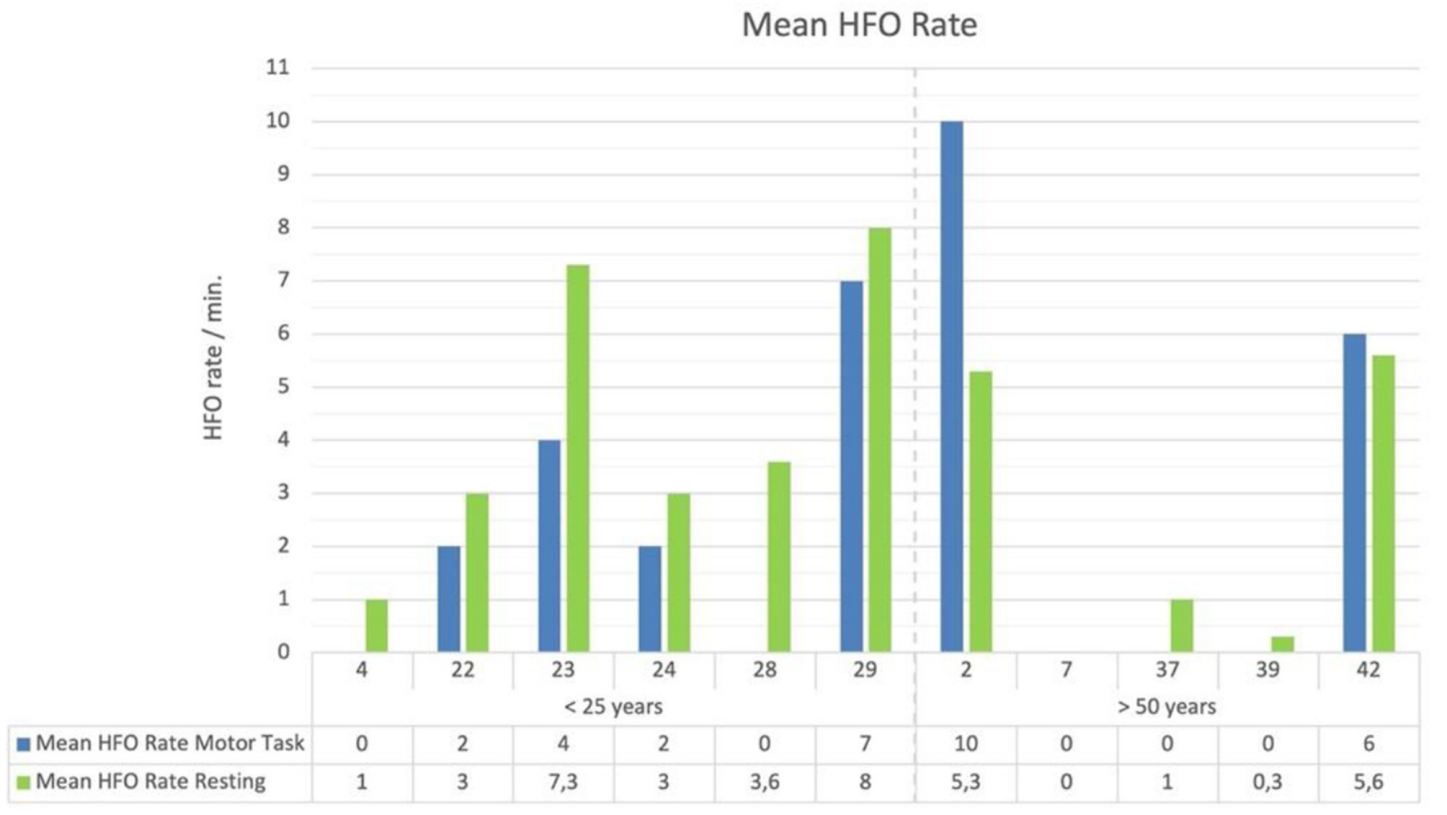
Mean HFO rate split up for tasks and age-groups.

Since the analyzed EEG segments differed between the tasks (resting vs. motor-task) with respect to their length (resting: 198 sec.; motor-task: 60 sec.), the HFO rate per minute was calculated for each patient. Figure 4 provides an overview about mean HFO rate of patients.

A control group was included to test for significant differences between patients suffering from epilepsy and patients with no epilepsy diagnosis. The control group was composed of EMU patients whose clinical picture was determined not to be a form of epilepsy. *Mann-Whitney tests* were carried out, comparing the ripple rates and the overall HFO rates both during the motor learning task and during resting phases. Because of multiple comparisons, all results were interpreted at the Bonferroni corrected level of significance *p*= 0.05/6 = 0.008.

To investigate a possible influence of sleep on mean HFO rate, we calculated a correlation between the two variables. The amount of sleep was based on patients' information about the hours they have slept the night before the testing.

To investigate a possible lateralization effect, we calculated correlations between the patients' age and the number of HFOs detected in the epileptic hemisphere, and in the non-epileptic hemisphere, respectively. Furthermore, we also calculated correlations between the patients' age and the number of HFOs detected during the motor task, and during resting phases, respectively. Because of multiple comparisons, all results were interpreted at the Bonferroni corrected level of significance *p* = 0.05/4 = 0.0125.

## Results

### Difference of HFO Rate; Young vs. Elderly Patients

The analyses were conducted separately for ripples, and ripples including unclear HFOs. Regarding the events detected while patients rested, a Mann-Whitney test indicated that there was no difference in the number of ripples between the younger and the older group of patients (*U* = 10.5, *p* = 0.429, *r* = 0.3).

Even after including unclear HFOs in the analysis, the Mann-Whitney test indicated that there was no difference between the two patient groups regarding the mean HFO rate during resting (*U* = 8.5, *p* = 0.247, *r* = 0.36).

During the learning trials of the fingertapping task, no clear ripple was identified in any of the patients. Regarding overall HFOs (*ripple* + *uHFO*), again the Mann-Whitney test indicated that there was no difference between the younger and the older group of patients (*U* = 14.0, *p* = 0.931, *r* = 0.06).

**Figure 5:**
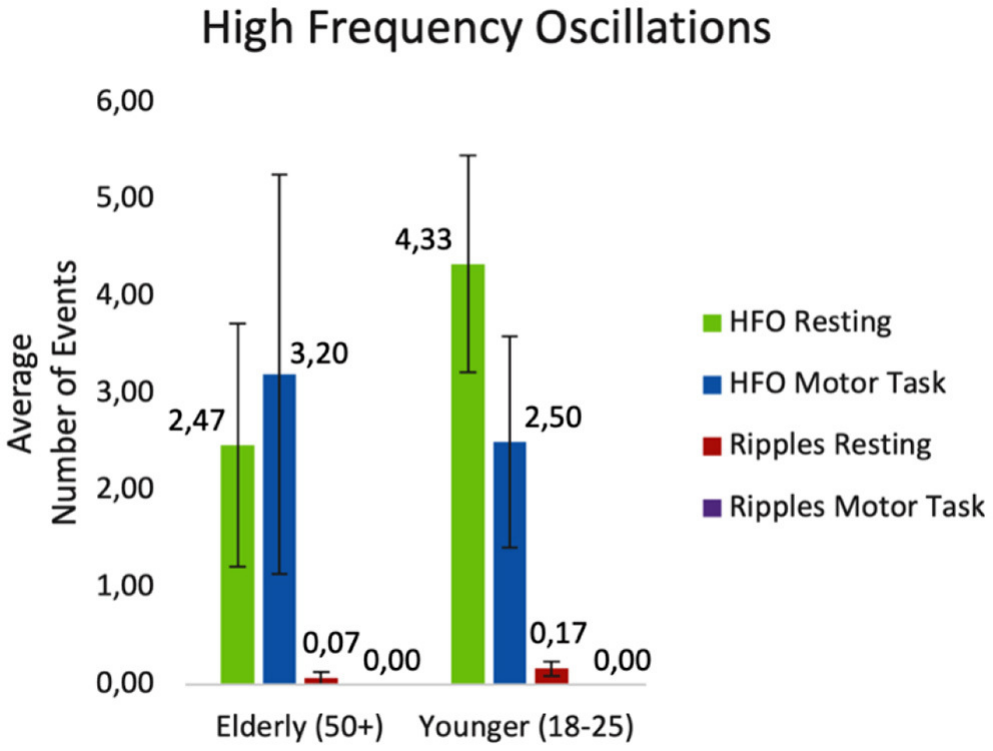
Average number of events detected while resting and during the motor task. HFO, ripples and unclear HFOs combined; Ripples, HFOs at 80–250 Hz.

The average number of detected events during resting and during the motor task are displayed in Figure 5.

Statistical analysis was based on the mean HFO rate of patients. The results of the semi-parametric repeated measures ANOVA revealed no significant difference for the factor age (*F*_(1, 25.53)_ = 0.09; *p*= 0.767). There was also no significant difference in HFO rate between conditions (*F*_(1, Inf)_ = 0.93; *p* = 0.334). Finally, the results revealed a significant interaction effect between the two groups during the tasks (*F*_(1, Inf)_ = 5.09; *p* = 0.024). During the motor tasks, more HFOs were identified in the elderly group, while the opposite was the case during resting, with more events detected in the younger group of patients.

### Difference of HFO Rate; Epilepsy Patients vs. Control Group

Regarding the events detected while patients rested, a *Mann-Whitney* test indicated that there was no difference in the number of ripples between the group of patients with epilepsy vs. the group without epilepsy (*U* = 23.0, *p* = 0.661, *r* = 0.16).

Even after including unclear HFOs the analysis, the *Mann-Whitney* test indicated that there was no difference between the two groups (*U* = 27.5, *p* = 1.00, *r* = 0.0).

No ripples were identified during the learning trials of the fingertapping task. Regarding overall HFOs (*ripple* + *uHFO*), the *Mann*-*Whitney* test indicated that there was no difference between the two groups (*U* = 26.0, *p* = 0.913, *r* = 0.04).

### HFO-Rate and Sleep

A Spearman's rank-order correlation was run to determine the relationship between the amount of sleep the patients had the night before the recording and the number of detected events, both while resting and during the motor task. There was no significant correlation between sleep and the number of detected HFOs while patients rested (r_s_(9) = −0.002, p = 0.995). The correlation between sleep and the number of detected HFOs during the motor task was also not significant (r_s_(9) = −0.300, *p* = 0.369).

### HFO-Rate and Age; Lateralization

Spearman's rho correlation coefficient was used to assess the relationship between the patients' age and the overall number of HFOs detected in the epileptic hemisphere, i.e. number of HFOs combined across the two conditions, motor task and rest. There was no significant correlation between the two (*r*_*s*_ = −0.397, *p* = 0.436). Furthermore, after correction for multiple comparisons there was also no significant correlation between the patients' age and the number of HFOs detected in the non-epileptic hemisphere (*r*_*s*_ = −0.841, *p* = 0.036).

To provide an overview about a possible hemispheric dominance of HFO activity, we created a table, displaying the epileptic hemisphere as diagnosed by the physician, as well as the total number of HFOs split up by their hemispheric occurrence. Events were merged across conditions of motor and rest, as there was also no significant main effect for condition. Furthermore, there was also no significant relation between the number of HFOs observed in the HD-EEG and the epileptic hemisphere diagnosed by the physician. However, when considering the data shown in Table 4, the relatively low numbers of HFOs must be taken into account.

**Table 4:** Diagnosed epileptic hemisphere and absolute HFO activity.

**ID**	Epileptic hemisphere^*^	**HFOs – Total number**	
		**Left hemisphere**	**Right hemisphere**
* <25 years*			
004	Right	2	0
022	Right	4	7
023	Left	14	11
024	Right	5	5
028	Front	6	5
029	NFD	11	15
*> 50 years*			
002	Left	6	3
007	Left	0	0
037	NFD	1	2
039	NFD	0	1
042	NFD	13	8

* *As diagnosed by the responsible physician; NFD, no final diagnosis*.

### HFO-Rate and Age; Motor Related HFOs vs. Resting HFOs

Spearman's rho correlation coefficient was used to assess the relationship between the patients' age and the number of HFOs detected during the motor task, and the number of HFOs detected during the resting phase, respectively. There was no significant correlation between the patients' age and the number of HFOs detected during performance of the motor task (*r*_*s*_ = −0.358, *p* = 0.486). There was also no significant correlation between the patients' age and the number of HFOs detected during the resting phase (*r*_*s*_ = −0.522, *p* = 0.288).

## Discussion

Because HFOs are intended to be used as a diagnostic marker for the identification of the seizure onset zone (SOZ) and ideally also for the epileptogenic zone in patients with epilepsy, we posed the question whether HFO occurrence on the scalp needs to take into account age as a confounding factor. We claim that examining the relationship between age and HFO rate might be crucial in developing an accurate diagnostic biomarker. Through the investigation of HFO rates in both elderly as well as young patients, we examined a possible change in HFO activity that comes with age. Therefore, we formulated the hypothesis that the HFO rate is increased in older populations and that it can be detected using scalp-EEG.

However, we were not able to replicate the general relation between HFO rate and age with the data collected in this study. HFO rate was not significantly elevated in an older population of patients across tasks. We may critically ask the question whether HFO occurrence is only elevated due to aging in prior research, or whether there are also factors of young age that contribute to HFO occurrence that were so far ignored. Our data showed an interaction of HFO rate by task, which we could interpret as a sign for age-related processing differences with respect to motor tasks. The difference between tasks in the older group is very small, while the younger group showed more HFOs during rest. Future studies with larger samples that are recruited with a broad distribution in age could shed more light on the age dependency in both the younger and older sub-group and assess these differences under different conditions and task requirements.

The sample is, however, still so small such that important moderators as the duration of epilepsy, medication, and localization of the seizure onset zone could not be considered in the analysis. Future studies with larger sample sizes, including also a critical number of healthy controls, are warranted in order to address this question.

Regarding patients' amount of sleep, we could not find a link between HFO event rate and the amount of sleep the night before the hospitalization. According to recent reports about influence of sleep on mean HFO rates, future studies need objective methods for the examination of sleep to rule out limitations like biases of self-disclosure. Additionally, increased pre-hospitalization nervosity that may influence sleep-pattern and therefore HFO activity should be considered.

Due to the small sample size and the heterogenous diagnoses it was not possible to perform an analysis by epilepsy type or epileptic focus. Nonetheless, we performed correlation between HFO rates found in the epileptic hemisphere as well as the non-epileptic hemisphere. This analysis revealed no lateralization effect for mean HFO rate, although a tendency for a significant correlation between age and HFO rate could be observed for the non-epileptic hemisphere. However, this effect was not significant after correction for multiple comparisons and the sample for this sub-analysis is rather small (*n* = 6), as for the other patients there was no clear localization of the seizure onset zone available. The rather high correlation coefficients warrant replication of this analysis in a larger sample. Furthermore, as can be seen in Supplementary Table 2, there is no clear pattern related to the type of epilepsy in terms of HFO rate.

Our results might contradict previous findings, but limitations and challenges regarding scalp HFO detection need to be considered. In contrast to Nakano and colleagues (44), who found that in invasive EEG the latter part of single HFO is enhanced in older subjects, using scalp EEG we investigated the influence of age on the total number of events occurring when performing different tasks rather than the increase of isolated segments of single events. Thus, our study design differs with respect to both the recording method and the primary objective. Although we did not demonstrate an age effect, the study at hand provides relevant considerations of how age associated aspects as well as non-age associated factors may influence validity and therefore quality of studies in the field.

In the following we will discuss the special setting of HFO detection in the scalp EEG that might explain the non-replicability of a relation between HFO rate and age in our sample.

### Signal to Noise Ratio

High- frequency somatosensory evoked potentials (HF-SEPs) have been the target of recent investigations examining focal epilepsy as a network disease rather than an isolated dysfunction of narrow cortical regions (55). It was found that these HF-SEP's had a significantly longer duration in the affected hemisphere compared to the unaffected hemisphere as well as compared to controls, therefore reflecting interictal functional impairments of the thalamo-cortical network. A study conducted by Nakano and Hashimoto (44) used invasive EEG to record somatosensory evoked potentials by median nerve stimulation. They found that the later part of HFO activity is increased in older patients. These invasive methods provide the advantage of a higher SNR, for example by minimizing artificial distortions due to cranial or facial muscle-movement. Additionally they show a higher sensitivity for small electrical signals arising from deep brain-regions. This explains the better identifiability of HFOs in the invasive EEG. In the scalp-EEG, a low background level of noise is indispensable for the identification of these correlates of small generators of neural signals (38). It is not always possible to keep the background noise level low, such that the identification of small events is less likely. In addition, artifacts may overlap with genuine HFOs. It has been claimed that the use of automatic detection would help to overcome this problem by valid identification of small events even in data with a higher background noise like it can be seen in scalp-EEG (56). Additionally, it has been assumed by previous studies that HFO-occurrence is in general rare in scalp EEG (38) which would be an alternative explanation of the lower HFO rates found in the actual study.

### Epilepsy vs. No-Epilepsy

In the study at hand, we were unable to find significant differences in HFO rates between patients suffering from epilepsy and patients with no epilepsy diagnosis. It has been shown by previous studies that HFOs are positively correlated with epileptic spikes arising from structural brain changes like they are observed in patients suffering from epilepsy (28). The fact that invasive EEG is almost exclusively conducted in patients with severe epilepsy could partly explain why some studies with invasive recordings report a higher rate of HFOs compared to our study, which exclusively analyzed scalp-EEG. Notwithstanding that HFOs can also be recorded in healthy subjects, they are less prominent than pathological ones. This was shown by Kandel and Buzsáki (57) where stimulation of mesial brain areas like the thalamus induced neocortical ripples. Because the study at hand was conducted on patients admitted to the EMU for epilepsy clarification it is possible that we recorded data from patients with less severe forms of epilepsy resulting in lower rates of identified HFOs.

Furthermore, since the study procedure was conducted on the first day of hospitalization before drug tapering was initiated, an influence of medication on the occurrence of HFOs cannot be ruled out. Unless otherwise stated, all patients in the study responded to their medication and did not suffer any form of drug resistant epilepsy. Out of 11 patients 4 had no medication at all. For a detailed overview about patients' medication see Supplementary Table 1. Studies in intracranial EEG show that medication withdrawal increases the rate of HFOs (58). Another study found that HF-SEPs are modulated by antiepileptic drugs (59). This study found that antiepileptic drugs reduce the amplitude as well as the duration of HFOs of the affected hemisphere. The HFO suppressing effects reported by these studies might explain why we were not able to find a significant difference in HFO activity between groups. Also, controlling for medication and other medical conditions statistically was not possible due to the small sample size and must be considered a limitation of the study.

### Sample Size & Power

The probability of being able to statistically detect a true effect depends on the statistical power of the study which in turn depends on the effect size and the sample size. Given the small average effect size reported in neuroscience in general and in EEG assessment in particular, the importance of an appropriate sample size cannot be stressed enough to increase reproducibility of studies (60).

Because relatively few studies investigated a relationship between HFOs and age in the scalp- EEG and the fact that almost no study offers information about the presumed effect size and how the sample size was calculated (61), there is nearly no data for orientation. To overcome the limitations of small and heterogenic samples multicentered studies are needed in the field of HFO research. This is very challenging due to missing standardized protocols for both, recording as well as for the analysis of data.

### Limitations of Segment Duration Using Scalp-EEG

A limiting factor regarding segmental length chosen in the study at hand was the time expenditure related to the manual analysis of the HD-EEG data. Due to this as well as an a priori determined study-duration and the goal of screening as many different patients as possible, we decided to analyze shorter EEG- segments of different tasks. Striving for highest possible quality, the study at hand aimed to map a maximal representative section of the entire EEG of the respective patients. In order to do so care was taken to achieve a maximum of data quality as well as a minimum of artificial bias. To overcome these shortcomings, future studies using automatic detection are needed therefore enabling the analysis of longer EEG-segments with the same or even less time expenditure.

### Pathological and Physiological HFOs

An important challenge of HFO research is the distinction of physiological and pathological HFOs. The idea of contrasting resting EEG with EEG during task performance in our study was set out to provoke physiological HFOs related to movement, and potentially distinguish them from pathological HFOs. However, it is still not possible to distinguish HFOs on a single-event basis and a very sophisticated design with provocative conditions might be needed to document increased HFO occurrence over regions that are functionally involved in execution of tasks and evoke physiological activity in the high frequency range.

Although we did not find significant differences in HFO activity between older and younger patients with respect to epileptic lateralization, it is noteworthy that the *p-*values in point 3.4 are rather small when comparing the non-epileptic hemispheres (r_s_ = −0.841, *p* = 0.036). There are 2 possible explanations for the actual *p*-values. On one hand, it could be that younger patients showed increased physiological HFO activity in the non-epileptic hemisphere compared to the older patients due to higher rate of neuronal activity. However, whether these HFOs were of pathological or physiological origin cannot be assessed in this study. On the other hand, it could be a purely coincidental finding due to the sample size. In favor of this theory would be the fact that, on close inspection of Table 3, there is no visible difference between the two hemispheres with respect to their HFO activity, which rather suggests against excessive physiological HFO activity. Although the Bonferroni correction provides some protection against false positives, it also poses the risk of creating false negatives. This should be taken into account when it comes to interpretation of the results.

The relevance of being able to distinguish between pathological and physiological HFOs also becomes apparent when it comes to the interpretation of the ANOVA. From a physiological point of view, an increased activity during motor tasks in older subjects could indicate an increased cognitive effort, whereas in younger subjects it could indicate a possibly higher consolidation activity in the resting phases associated with the maintenance of the numerical sequences of fingertapping. From a pathological point of view, higher HFO rates could be indicative of neurodegenerative processes in task associated areas in older subjects, with a decreased activity in younger patients.

However, the most likely explanation in this case is that it is a coincidental finding due to the rather small sample size.

Although this study included various conditions, it is not easily possible to distinguish physiological from pathological HFOs with this study design. Interestingly, especially HFOs recorded during sleep can be highly indicative in these respects, as recently shown (61). Specifically, HFOs recorded with invasive EEG during sleep were described to occur mainly in the hippocampus and occipital lobe, irrespective of the seizure onset zone or lesions related to epilepsy (62). We did not have HD-EEG recordings during sleep at our disposal. Future studies should investigate the possibility to record physiological high frequency activity during deep sleep. However, we investigated resting phases before and between our tasks regarding HFO activity. Although this is not a substitute for long-term recordings it still serves the purpose of capturing an interictal activity with increased signal-to-noise ratio.

## Conclusions

In this study, we did not confirm a difference in HFO-rate between a young (<25 years) and older (>50 years) group of patients with epilepsy. Several reasons may account for the non-significant differences between age groups observed in the scalp-EEG and future studies should aim at a larger sample size, analysis of longer EEG segments, acquisition of sleep-EEG, testing under various conditions of rest and cognitive effort, and record under drug withdrawal. Because HFO detection in scalp EEG provides a cheap and non-invasive alternative to invasive EEG it is important that further studies investigate if an age dependent increase of HFO activity is associated with structural aging processes rather than being of epileptogenic nature. We deem it to be crucial to take into account biases such as age when introducing HFOs as a new biomarker in the diagnosis and management of epilepsy.

## Data Availability Statement

The datasets presented in this article are not readily available because of privacy issues. Requests to access the datasets should be directed to philipp.windhager@hotmail.com.

## Ethics Statement

The studies involving human participants were reviewed and approved by Ethik-Kommission für das Bundesland Salzburg: E/1755, 415-E/1806/4-2014, initial approval on 30/03/2014, latest amendment on 11/07/2016. The patients/participants provided their written informed consent to participate in this study.

## Author Contributions

YH: conceptualization, writing—review, editing, project administration, and funding acquisition. PW, AM, and YH: methodology and writing—original draft preparation. PW and AM: validation, investigation, and visualization. AM: formal analysis and data curation. ET and AB: resources. YH, ET, and AB: supervision. All authors have read and agreed to the published version of the manuscript.

## Funding

This research was funded by the Austrian Science Fund (FWF), grant number KLI 657-B31, and by the Research Fund of the Paracelsus Medical University (PMU-FFF). The APC was funded by the Austrian Science Fund (FWF) and by the Research Fund of the Paracelsus Medical University (PMU-FFF), grant number A-18/01/029-HöL.

## Conflict of Interest

The authors declare that the research was conducted in the absence of any commercial or financial relationships that could be construed as a potential conflict of interest.

## Publisher's Note

All claims expressed in this article are solely those of the authors and do not necessarily represent those of their affiliated organizations, or those of the publisher, the editors and the reviewers. Any product that may be evaluated in this article, or claim that may be made by its manufacturer, is not guaranteed or endorsed by the publisher.
