# Supplementary material for: Are High Frequency Oscillations in Scalp EEG Related to Age?
Source: Front Neurol. 2022 Jan 27;12:722657. doi: 10.3389/fneur.2021.722657 (PMC8829347; doi:10.3389/fneur.2021.722657)
Supplement: Supplementary file 1 [file Table_1.DOCX]

| ID | Sex | Age | MRI Evidence | EEG Evidence | Medication | Epilepsy | Seizure Type | |
| --- | --- | --- | --- | --- | --- | --- | --- | --- |
| 004 | F | 24 | Hippocampal malrotation (left) | Ictal: 1. bifrontal alpha activtiy 2. rhythmic theta activity (left temporal); Interictal: intermittent theta activity (left temporal) | Levetiracetam (1500mg - 0 - 1500mg - 0); Vimpat (200mg - 0 - 200mg -0); Perampanel (0-0-0-4mg) | TLE right | Focal aware and focal to bilateral tonic-clonic seizures | |
| 022 | F | 23 | Hippocampal sclerosis (right) | Ictal: 1. SOZ right temporal region 2. also two seizures with a predominantly left ictal pattern; Interictal: discrete pathologies right hemisphere | Lamotrigin 500mg | MTLE right | Focal impaired awareness seizures | |
| 023 | F | 22 | Lesion - fronto-parietal to left occipital area | SEEG - Ictal: SOZ left occipital region; Interictal: irritative zone left occipito-parietal region | Levetiracetam (750mg-0-750mg-0); Lamotrigin (75mg-0-75mg-0) | PTME Left | Focal aware seizures; Focal impaired awareness seizures; Focal to bilateral tonic-clonic seizures | |
| 024 | M | 19 | Discreet changes to the occipital cortex (right); suspect of ulegyria | Ictal: SOZ right parieto-occipital region; Interictal: bilateral theta activity in the parieto-occipital region | Levetiracetam (1000mg-0-1000mg-0); Vimpat (200mg-0-200mg-0) | OLE Right | Focal aware seizures; Focal impaired awareness seizures; Focal to bilateral tonic-clonic seizures |  |
| 028 | M | 19 | Gliosis / Transmantle Sign | normal / uneventful | None | FLE | Focal aware seizures; Focal impaired awareness motor seizures; Focal to bilateral tonic-clonic seizures | |
| 029 | F | 22 | Non-lesional | no ictal events; interictal: single steep transient in the left basal temporal area | Lamotrigin 25 mg 1-0-1 | NFD | Focal to bilateral tonic- clonic seizures | |
| 002 | M | 56 | N/A | TIRDAs; no spikes or sharp waves | none | TLE left | Focal aware seizures | |
| 007 | F | 59 | Non-lesional | normal / uneventful | Levetiracetam 500 mg (1/2-0-1/2-0) | TLE left | Focal impaired awareness seizures | |
| 037 | M | 64 | N/A | normal / uneventful | Lamotrigin 150mg (50mg-0-100mg) | NFD | Focal to bilateral tonic- clonic seizures | |
| 039 | F | 54 | Non-lesional | normal / uneventful | None | NFD | Speech impairment; cognitive restrictions; vertigo | |
| 042 | M | 55 | Bifrontal corticomedullar signal increase | normal / uneventful | none | NFD | Focal to bilateral tonic- clonic seizures | |

M: Male; F: Female; N/A: not available; NFD: no final diagnosis; TLE: Temporal Lobe Epilepsy; MTLE: Mesial Temporal Lobe Epilepsy; PTME: Posttraumatic Multifocal Epilepsy; OLE: Occipital Lobe Epilepsy; FLE: Frontal Lobe Epilepsy

**Supplementary Table 1.** Detailed patient information

| ID | Sex | Age | Epilepsy | Resting HFO | Motor HFO |
| --- | --- | --- | --- | --- | --- |
| < 25 Years |  |  |  |  |  |
| 004 | F | 24 | TLE right | 3 | 0 |
| 022 | F | 23 | MTLE right | 9 | 2 |
| 023 | F | 22 | PTME Left | 22 | 4 |
| 024 | M | 19 | OLE Right | 9 | 2 |
| 028 | M | 19 | FLE | 11 | 0 |
| 029 | F | 22 | NFD | 24 | 7 |
| > 50 Years |  |  |  |  |  |
| 002 | M | 56 | TLE left | 16 | 10 |
| 007 | F | 59 | TLE left | 0 | 0 |
| 037 | M | 64 | NFD | 3 | 0 |
| 039 | F | 54 | NFD | 1 | 0 |
| 042 | M | 55 | NFD | 17 | 6 |

M: Male; F: Female; NFD: no final diagnosis; TLE: Temporal Lobe Epilepsy; MTLE: Mesial Temporal Lobe Epilepsy; PTME: Posttraumatic Multifocal Epilepsy; OLE: Occipital Lobe Epilepsy; FLE: Frontal Lobe Epilepsy.

**Supplementary Table 2.** Epilepsy and HFO rates.
